# Natural *versus* Recombinant Viral Antigens in SARS-CoV-2 Serology: Challenges in Optimizing Laboratory Diagnosis of COVID-19

**DOI:** 10.6061/clinics/2020/e2290

**Published:** 2020-11-26

**Authors:** Luciana Regina Meireles, Angélica Moura Freixeira da Silva, Camila Aparecida Carvalho, Norival Kesper, Andrés Jimenez Galisteo, Camila Pereira Soares, Danielle Bastos Araujo, Edison Luiz Durigon, Danielle Bruna Leal Oliveira, Lígia Morganti, Rosa Maria Chura-Chambi, Heitor Franco de Andrade

**Affiliations:** I Laboratorio de Protozoologia do Instituto de Medicina Tropical de Sao Paulo, Faculdade de Medicina (FMUSP), Universidade de Sao Paulo, Sao Paulo, SP, BR.; II Departamento de Microbiologia, Instituto de Ciencias Biomedicas da Universidade de Sao Paulo (ICB-USP), Sao Paulo, SP, BR.; III Instituto de Pesquisas Energeticas e Nucleares (IPEN-CNEN/SP), Centro de Biotecnologia, Sao Paulo, SP, BR.; IV Plataforma Cientifica Pasteur - USP, Sao Paulo, SP, BR.

**Keywords:** COVID-19, SARS-CoV-2, Serological Test, ELISA, Recombinant Nucleocapsid Protein, SARS-CoV-2 Whole Viral Antigen

## Abstract

**OBJECTIVES:**

COVID-19 is a public health emergency of international concern whose detection in recovered asymptomatic patients is dependent on accurate diagnosis as it enables the estimation of the susceptibility of the population to the infection. This demand has resulted in the development of several commercial assays employing recombinant proteins, but the results of these assays are not reliable as they do not involve comparison with natural viral antigens. We independently used the SARS-CoV-2 whole viral antigen (WVA) and recombinant nucleocapsid protein (rNP) to develop in-house ELISAs for IgG detection; the results of these ELISAs were then compared to obtain reliable results.

**METHODS:**

WVA and rNP ELISAs were performed on COVID-19 negative sera from patients before the pandemic in Brazil, and on RT-qPCR-positive or SARS-CoV-2-IgG against rNP and IgG against WVA-positive samples from recently infected patients in Sao Paulo, Brazil.

**RESULTS:**

Both ELISAs detected a large fraction of infected patients but exhibited certain drawbacks. Higher signals and lower numbers of false-negatives were observed in rNP ELISA; however, a higher fraction of false-positives was observed in control groups. A high number of false-negatives was observed with WVA ELISA. Correlating the results of rNP and WVA ELISAs resulted in improved performance for COVID-19 diagnosis.

**CONCLUSION:**

The choice of antigen is an important aspect in optimizing the laboratory diagnosis of COVID-19. The use of rNP ELISA for the detection of anti-SARS-CoV-2 IgG antibodies seems promising, but comparison of the results with those of WVA ELISA is crucial for accurate test development prior to commercialization. IgG serology using several assays, and with the spectral patterns of SARS-CoV-2, resulted in confusing information that must be clarified before the establishment of diagnostic serology criteria.

## INTRODUCTION

A novel and highly transmissible human coronavirus (SARS-CoV-2) was identified as being responsible for severe acute respiratory syndrome: Coronavirus disease 2019 (COVID-19). The disease was declared by the World Health Organization as a pandemic, and several molecular and serological tools have been developed to identify ongoing viral infections and to evaluate immunity against SARS-CoV-2 (1-4,6,7). The diagnosis of SARS-CoV-2 infection involves collecting the correct specimens from patients at the right time. Molecular tests have been used to detect acute cases of infection, while serological tests have been used to identify individuals who have developed immunity against SARS-CoV-2 (2,3).

SARS-CoV-2 detection using real-time reverse transcriptase polymerase chain reaction (RT-qPCR) is considered the ‘gold standard’ for COVID-19 diagnosis (2,3). RT-qPCR is used in the diagnosis of COVID-19 in patients with respiratory illness, as well as for screening the contacts (3). SARS- CoV-2 can be detected in upper respiratory samples 1-2 days prior to the onset of symptoms, and can persist for 7 to 12 days in moderate cases, and up to 2 weeks in severe cases (8). However, this technique requires certified laboratories, expensive equipment, and trained technicians, and can yield false-negative results (9,10). Even though the analytical sensitivity of this technique is generally known to be very high, detection is dependent on several crucial factors such as sampling timing related to disease onset, sample quality, transport, and storage conditions (3,11). RT-qPCR may not detect the virus in the very early or the very late stages of infection when the viral load is very low (12).

Serological tests for the detection of specific antibodies against SARS-CoV-2 in blood samples remain a good choice and alternative for diagnosing COVID-19. These tests offer a number of advantages over RT-qPCR. They are less expensive than molecular tests, require shorter analytical time and processing, and can offer much greater throughput than automated molecular tests (3,5,6). In addition, human antibodies are known to be more stable than viral RNA. Consequently, serological samples are less prone to deterioration than RT-qPCR samples during sample collection, preparation, transport, storage, and testing. Furthermore, serological samples exhibit fewer variations than nasopharyngeal specimens because antibodies are usually homogeneously dispersed in the blood (3). Serological tests can also provide additional details as they can be used to identify individuals who have developed virus-specific antibodies, thereby providing better information regarding disease prevalence in a population. In the near future, serological tests could also be used to evaluate vaccine efficacy (6,13).

Antibody dynamics for COVID-19 was found to be similar to that of acute viral infections in which IgG levels increase at the time IgM levels start to decrease (7,14). It is estimated that SARS-CoV-2 IgM antibodies can be detected in a blood sample 3-6 days after the onset of symptoms and IgG antibodies can be detected 8 days after the onset of symptoms (3,15). Serological assays designed to detect IgM/IgG antibodies are important for diagnosing COVID-19, and can help in developing a better understanding the burden imposed by asymptomatic infections (1).

Although high-throughput testing has been proposed to reduce the burden imposed by COVID-19, meta-analyses on the accuracy of commercially available immunological tests for SARS-CoV-2 reveal that the performance of these tests remains uncertain. According to Caini et al. (4), random-effects models yield a summary sensitivity of 82% for IgM and 85% for IgG and total antibodies. For specificity, the pooled estimate was 98% for IgM and 99% for IgG and total antibodies. Similarly, a systematic review and meta-analysis by Bastos et al. (5) revealed the sensitivities of enzyme-linked immunosorbent assays (ELISAs) for measuring IgG or IgM levels, lateral flow immunoassays (LFIAs), and chemiluminescence immunoassays (CLIAs) to be 84.3% (95% CI: 75.6% to 90.9%), 66.0% (95% CI: 49.3% to 79.3%), and 97.8% (95% CI: 46.2% to 100%), respectively. Investigations regarding the accuracy of commercially available tests for COVID-19 in Brazil revealed unclear data. Castro et al. (2) analyzed the diagnostic accuracy of 16 commercial assays registered by the Brazilian Health Regulatory Agency (ANVISA). The sensitivity of the tests—registered by the Brazilian health regulatory agency—for IgM antibodies was 82% (95% CI: 76% to 87%), and the specificity was 97% (95% CI: 96% to 98%). For tests developed to detect IgG antibodies, the sensitivity was 97% (95% CI: 90% to 99%) and specificity was 98% (95% CI: 97% to 99%). Although the specificity of the tests was generally high (≥ 98%), it may not suffice to guarantee satisfactory performance in areas with a very low number of infected individuals, particularly if there is a large proportion of asymptomatic individuals (4).

Differences between the accuracy of the tests can be attributed to several factors, including the nature of the viral antigen(s) used for antibody detection and the type of antibodies identified. Unfortunately, the majority of diagnostic companies do not report the nature of the antigens utilized in their assays, and it is therefore difficult to compare results. The most common viral proteins used as antigens in the available serological assays are recombinant proteins including nucleocapsid protein (N), transmembrane spike protein (S), or protein S subunits (6,16). The detection of antibodies directed against spike protein or its subunits is more likely to reflect neutralizing antibody effects, and would better demonstrate the immunity status, on the other hand, assays employing the N protein generally show better sensitivity (4). To the best of our knowledge, kits containning the whole viral antigen (WVA) of SARS-CoV-2 are not commercially available. In the past, most serological tests for viral diseases such as measles (17) and rubella (18), have been performed using natural viral proteins from the infected cell cultures. Although the production of these antigens is laborious because of the need for cell culture and adequate biosafety conditions, the use of WVAs instead of recombinant proteins in serological tests, could result in a better diagnostic accuracy for viral infections (19). In the present study, we proposed to standardize an in-house ELISA based on comparison of the results of SARS-CoV-2 WVA and SARS-CoV-2 rNP ELISAs to assess whether the association of these different antigens could reduce variability in estimates and enhance the performance of ELISAs for the detection of anti-SARS-CoV-2 IgG in stratified groups of asymptomatic patients, and in individuals with mild or moderate symptoms. We utilized a 35 kDa fragment of rNP that lacked a highly conserved motif (residues 1-121) found in all coronaviruses, including SARS-CoV-2 (20), to minimize the occurrence of false-positives owing to the presence of antibodies against other coronaviruses. The use of WVA may be useful for future validation of candidate recombinant proteins in COVID-19 tests.

## MATERIAL AND METHODS

### Serum Samples

This study was performed using 111 serum samples, of which 50 were obtained from the biorepository collection of the Laboratory of Protozoology of the Institute of Tropical Medicine, University of Sao Paulo (IMT/USP) (São Paulo, Brazil). The other 61 samples were obtained from volunteers who were diagnosed with COVID-19 using molecular testing (RT-qPCR) and/or based on the presence of anti-SARS-CoV-2 IgGs. The biorepository collection samples were used as controls as they were collected before the COVID-19 pandemic. All procedures were approved by the Research Ethics Committee of Hospital das Clínicas, School of Medicine of the University of Sao Paulo (HCFMUSP) (São Paulo, Brazil) (protocol number: 31685020.4.0000.0068).

### SARS-CoV-2 antigen production

The SARS-CoV-2 strain (SARS-CoV-2/SP02/human/2020/BRA) used in ELISA in the present study was isolated and provided by Professor Edison Luiz Durigon of the Institute of Biological Sciences, University of Sao Paulo (ICB/USP) (São Paulo, Brazil). This strain was isolated from the first two identified cases of COVID-19 in Brazil (21). The virus was cultured in Vero E6 cells and maintained at 37 °C in Dulbecco’s minimal essential medium (DMEM) (Vitrocell Embriolife, Campinas, SP, Brazil) supplemented with 2% heat-inactivated fetal bovine serum (FBS) (Vitrocell Embriolife, Campinas, SP, Brazil), as described by Araujo et al. (21). When the cells exhibited 90% cytopathic effects (CPE), 1% formaldehyde and 1% hydrogen peroxide were added to the culture medium for viral inactivation. After 12 h, the media and cell layers were harvested for viral antigen production.

### WVA from cell layers

After viral inactivation, the media and cell layers were harvested and cleared by centrifugation at 10,000 × *g* at 4 °C for 15 min, and the supernatant was carefully removed. The pellet was resuspended in lysis buffer pH 7.5 (0.05 M Tris/HCl, 0.075 M NaCl, 10 mM EDTA, 0.5% Sodium Desoxycholate (DOC), and 0.5% Sodium Dodecyl Sulphate (SDS)) and incubated at 65 °C for 30 min. The suspension was centrifuged at 10,000 × *g* at 20 °C for 15 min, and the supernatant containing WVA was stored at -80 °C until use.

### Recombinant nucleocapsid protein (rNP)

A 122-419 bp fragment of the gene that codes for SARS-CoV-2 nucleocapsid protein (PubMed Accession No. QIG 56001) with two histidine tags, one at the N- and another at the C-terminal, was synthesized by GenScript (Piscataway, New Jersey, USA) and cloned into the plasmid pET28a (Merck Millipore Inc) (Novagen, Darmstadt, Germany). rNP was expressed as inclusion bodies in *Escherichia coli*, strain BL21 (DE3), and refolding was performed at pH 9 and 2.4 kbar for 90 min, as described by Chura-Chambi et al. (22).

### SDS-PAGE and western blotting

SARS-CoV-2 antigens were resolved by sodium dodecyl sulfate polyacrylamide gel electrophoresis (SDS-PAGE) on 4-20% gradient gels. Briefly, 10 µL of WVA (0.1 mg/mL) and 10 µL of rNP (0.1 mg/mL) were diluted (v/v) in Laemmli sample buffer (Bio- Rad^®^, California, USA) and heated at 95 °C for 5 min. Electrophoresis was performed at room temperature (22 °C-24 °C) for approximately 45 min at a constant voltage (120 V) using PowerPac^TM^ HC High-Current Power Supply (Bio-Rad, Singapore) followed by electrophoretic transfer to nitrocellulose membranes (Sartorius Stedim Biotech, Goettingen, Germany) using Trans-Blot Turbo Transfer System (Bio-Rad, Singapore). The membranes were blocked with 5% fat-free milk prepared in Phosphate-Buffered Saline (PBS) containing 0.05% Tween 20 (PBST), and these membranes containing the antigens were incubated with serum samples diluted 1:100 for 18 h at 4 °C. After washing with PBST, bound antibodies in the serum samples were detected by incubation with peroxidase-conjugated anti-human-IgG for 1 h at room temperature (22 °C-24 °C), followed by washing and incubation with 3, 3'-diaminobenzidine (DAB) until visualization of protein bands. The reaction was stopped with distilled water.

### Enzyme-linked immunosorbent assay

Anti-SARS-CoV-2 IgG antibodies were detected using an in-house ELISA. Briefly, a 96-well microplate (Corning^®^, New York, USA) was coated with WVA obtained from cell culture and rNP (0.1 mg/mL) diluted in 0.1 M sodium carbonate buffer (pH 9.5). These antigens were assayed in separate wells (100 µL/well), and the microplates were maintained overnight at 4 °C in a humid chamber. The microplates were washed with PBST and blocked with 250 µL of 5% fat-free milk (Molico^®^, Nestlé, Araçatuba, Brazil) diluted in PBST for 1 h at 37 °C. After washing, 100 µL of serum samples diluted 1:100 in PBST containing 5% fat-free milk (PBST-milk) were incubated with viral antigens for 1 h at 37 °C. The microplates were again washed and 100 µL of anti-human IgG peroxidase conjugate (Sigma, St. Louis, USA) diluted 1:20,000 in PBST-milk was added per well and the plates were maintained for 1 h at 37 °C. After washing, 100 µL of 2, 2- azino-bis (3-ethylbenzothiazoline-6-sulfonic acid) - ABTS substrate solution) was added per well. The microplates were incubated in the dark for 30 min at room temperature (22 °C-24 °C). The reaction was stopped with 0.1 M citric acid (100 µL/well). The optical density (OD) at 414 nm was measured using a microplate reader (Labsystems Multiskan MS^®^, Midland, Canada). ELISA results were expressed in arbitrary units (AU) calculated by the ratio between the average absorbance of samples and the reaction cut-off.

### Statistical analysis

The cut-off threshold was determined using the receiver operating characteristic (ROC) curve. ROC and serological analyses (sensitivity, specificity, negative predictive value, positive predictive value, and likelihood ratio) were performed using GraphPad Prism 5.0 software (GraphPad Software, La Jolla, CA, USA). Frequencies were compared using bicaudal Chi-squared assays. Values when the probability of equality was less than 5% (*p*<0.05) were considered significant.

## RESULTS

### Characterization of viral antigens

**Figure 1:**
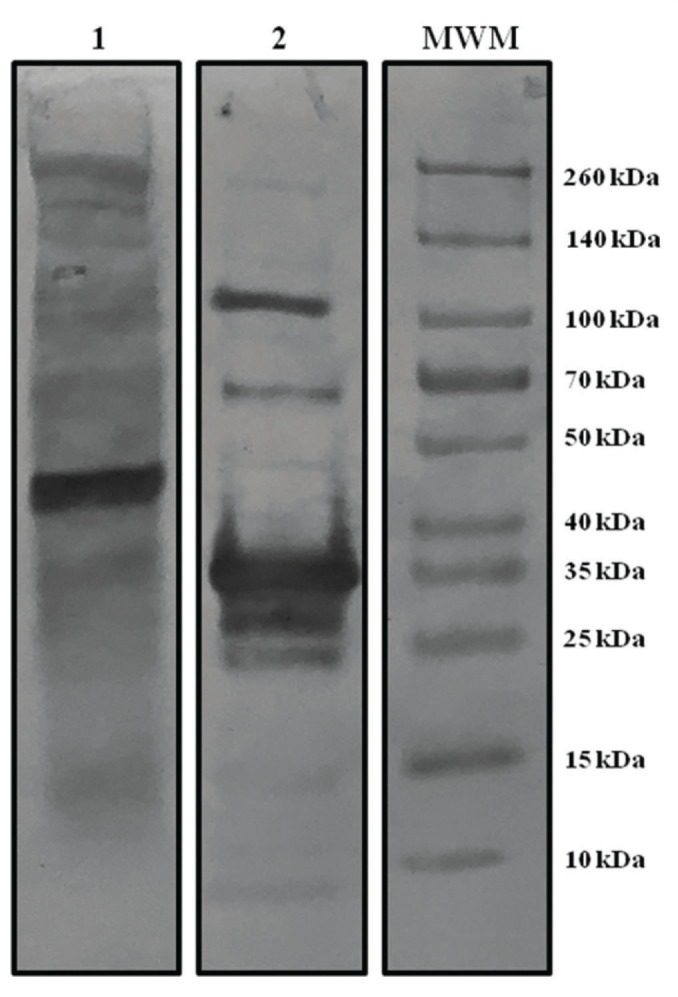
Digital images of protein patterns after western blotting using hyperimmune serum (from a patient with moderate symptoms) for the two ELISA antigens. Lane 1: Whole viral antigen pattern (WVA). Lane 2: Recombinant nucleoprotein (rNP) pattern. MWM: markers with known molecular weights.

Viral antigens used in ELISA were characterized by western blotting with a serum sample from a patient with moderate COVID-19 symptoms. Figure 1 shows the protein band pattern of WVA and rNP recognized by the hyperimmune serum. In case of WVA, several bands with specific reactivity for the spike protein (∼260 kDa) and nucleocapsid protein (45 kDa) were identified. rNP was detected as a specific band of 35 kDa, and this molecular weight corresponded to the expected molecular weight of our NP fragment.

### Enzyme-linked immunosorbent assay

**Figure 2:**
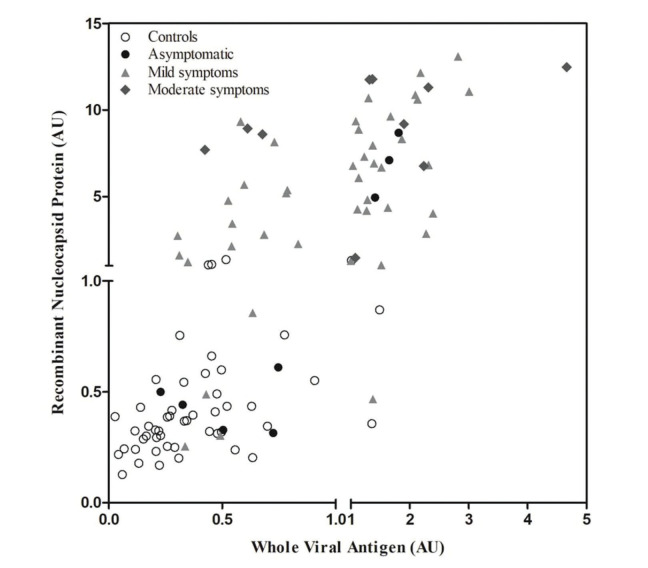
Correlation of ELISA results using whole viral antigen (WVA) and recombinant nucleocapsid protein (rNP) for control, asymptomatic and symptomatic groups. The results are expressed in arbitrary units (AU).

The summarized results of ELISAs using WVA and rNP are presented in Figure 2. The figure includes the data obtained for control samples collected before (n=50) and during (n=61) the pandemic, stratified based on the symptoms, *i.e.*, asymptomatic (n=8), mild symptoms (n=3), and moderate symptoms (n=10). Data distribution revealed that rNP ELISA was better at identifying the infected individuals; it yielded 10 false-negative and 4 false-positive results. WVA ELISA yielded 25 false-negative and 3 false-positive results. Although WVA ELISA exhibited lower sensitivity, it was able to identify one sample from the infected group which was not detected by rNP ELISA. WVA ELISA also correctly identified one sample from the control group that was incorrectly detected as an antibody by rNP. The sensitivity, specificity, positive predictive values, negative predictive values, and likelihood ratios for each antigen are presented in Table 1. We also demonstrated that the values obtained upon correlating the results of both tests resulted in better performance of our in-house ELISA.

The analyzed data revealed that the detection of IgG antibodies in control, asymptomatic, and moderate symptom groups was similar in case of both antigens. The rNP ELISA was able to identify a larger number of patients with mild symptoms, reacting with 76% of those sera (*p*<0.001), which were otherwise negative with WVA ELISA (Table 2).

## DISCUSSION

Conventional serological assays (ELISAs) for specific anti-SARS-CoV-2 antibodies can be an alternative—or complement—to real-time RT-qPCR in the diagnosis of COVID-19. The nature of the antigen is an important factor determining the performance of the serological tests, and our choice of the N protein instead of the spike protein can be justified by its generally high sensitivity. However, the latter is usually more likely to induce the production of neutralizing antibodies, and would better reflect immune status. In order to solve this issue, we evaluated whole viral proteins from SARS-CoV-2 cultured in Vero cells. This antigenic suspension presented the main SARS-CoV-2 proteins, including N and S, and allowed the detection of neutralizing antibodies and those against other viral proteins.

Correlating the results of rNP and WVA ELISAs improved the performance, and the specificity of our test was similar to that of commercially available tests in Brazil (2). In this study, rNP ELISA identified 4 samples from the control group as an antibody. A disadvantage of the whole nucleocapsid protein ELISA is that it can produce false-positive results as this protein is the most conserved viral protein among human betacoronaviruses (3,23). Nucleocapsid residues 1-121 were removed to minimize non-specific antibody reactions involving non-SARS-CoV-2 nucleocapsid antigens. However, it is possible that the 122-419 amino acid rNP and WVA antigens used in our ELISA could react with antibodies against other types of coronaviruses (HKU1, 229E, OC43, NL63) that are known to cause respiratory diseases (3,7,24). As rNP was >90% pure, it was not purified, and there is a possibility that the antibodies reacted with *E. coli* proteins.

Our data demonstrated that rNP ELISA showed greater sensitivity than WVA ELISA with respect to detecting infected individuals, mainly those in the mild symptoms group. Although less sensitive, WVA ELISA was able to identify one false-negative and four false-positive cases which were incorrectly detected using rNP ELISA. These data demonstrated the importance of using natural viral antigens to assess the performance of tests based on the use of recombinant proteins as antigens. The difference in sensitivity of our ELISAs may be attributed to the nature of the antigen used in each test. Although the natural viral antigen comprised different SARS-CoV-2 proteins, including nucleocapsid and spike proteins, the amount of these proteins in antigenic suspensions was proportionally lower than the recombinant protein used in our ELISA.

With respect to the sensitivity of the ELISA, correlating the results of rNP and WVA ELISAs resulted in a better diagnostic performance even though 9 samples were false-negatives. These data were similar to those reported by meta-analysis studies (5). The sensitivity of ELISAs for the detection of anti-SARS-CoV-2 IgG antibodies depends largely on the time of testing or on the day of illness. Antibody responses against SARS-CoV-2 virus may not be detectable in the early stages of infection. Although IgM antibodies may be generated rapidly owing to the presence of viral genetic material in the respiratory tract, the timing of immunoglobulin production (from 4 days after the onset of symptoms, to 10-14 days) limits its applicability in acute phase diagnosis (3,6,25,26,27). A study comparing the performance of serological tests with respect to the time of SARS-CoV-2 infection revealed the sensitivity for IgM and IgG detection to be 91.7% and 79.2%, respectively, in samples collected 7 days after PCR confirmation. The sensitivity of the assays for IgM and IgG detection increased to 100% in samples assayed 9 and 12 days after initial PCR confirmation (7). These data demonstrated that the use of serological methods for COVID-19 diagnosis requires the correct and appropriate interpretations of the results, and an understanding of the strengths and limitations of such tests, mainly with respect to the time of infection. These considerations are extremely important, and can help in the interpretation of our data, and reveal that the sensitivity of our ELISA could have been higher if we had used samples collected at a later period of infection, as most of them were collected 15 days after PCR confirmation.

It is important to highlight that recent studies demonstrate that individuals with mild symptoms of COVID-19 do not produce antibodies (3,28,29). It has been proposed that the innate immune system (cell-mediated immunity) wipes out the virus before the adaptive immune system produces antibodies (3,29). A number of studies have presented information regarding the immune response during SARS-CoV-2 infection, which involves antibody production and T lymphocyte activation. Most of this information was restricted to hospitalized patients, because they were infected but symptomatic (28). Over the course of the disease in hospitalized patients who recovered, antibody production was shown to increase after the first week of onset of symptoms (28,30), while T cell levels increased after 14 days of hospitalization (28). Interestingly, some people who presented positivity in the molecular test did not have detectable levels of protective antibodies (28). Furthermore, neutralizing antibodies were low or not present in hospitalized patients (28,30). A few studies have already shown that T cells might be key in solving this issue (28,30). Although SARS-CoV-2 can induce lymphopenia and cause a delay in T cell pathway activation during the first days of infection, after two weeks of symptoms, SARS-CoV-2-specific memory T cell phenotypes start to emerge in the peripheral blood, providing protective immunity (28).

Further data on description of antibody profiles in SARS-CoV-2 infection, presence of antibodies, their correlations with protective immunity, and duration of protection need to be explored. For all of these issues, serological tests may serve as a useful tool to improve COVID-19 diagnosis, and the WVA produced during viral infections of mammalian cells might be used for the validation of candidate recombinant proteins in COVID-19 tests.

## CONCLUSIONS

Our data demonstrated that the use of ELISA using rNP for the detection of anti-SARS-CoV-2 IgG antibodies is promising in individuals with mild or moderate symptoms of infection. However, the comparison of these assays with natural viral antigens is crucial for adequate development and must be performed before commercialization. Both ELISAs proposed in this study should be used with caution and in regions of low COVID-19 prevalence, since they can give false-negative results.

## AUTHOR CONTRIBUTIONS

All authors affirm that they have contributed to the development of the manuscript. Meireles LR designed the study, collected data, performed analysis, evaluated study findings, discussed results, and wrote the manuscript. Silva AMIF contributed to the investigation, methodology, and manuscript review. Carvalho CA contributed to the investigation, collection of blood samples from volunteers, and manuscript review. Kesper Junior N contributed to the investigation, contacted volunteers, and reviewed the manuscript. Galisteo Jr AJ contributed to the investigation and the manuscript review. Durigon EL, Soares CP, Araujo DB, and Oliveira DBL were responsible for supplying SARS-CoV-2 samples, and contributed to manuscript review. Morganti L and Chura-Chambi RM were responsible for producing rNP, and contributed to manuscript review. Andrade Jr HF contributed to the study design, performed analysis, verified the analytical methods, and provided support in the writing and editing of the manuscript.

## Figures and Tables

**Table 1:** Performance of WVA ELISA and rNP ELISA and performance after correlating the results of both ELISAs.

Parameters	Performance of WVA ELISA	Performance of rNP ELISA	Performance after correlating WVA/rNP ELISAs
Sensitivity	59.02% (95% CI 45.68%-71.45%)	83.61% (95% CI 71.91%-91.85%)	85.25% (95% CI 73.83%-93.02%)
Specify	94.00% (95% CI 83.45%-98.75%)	92.00% (95% CI 80.77%-97.78%)	98.00% (95% CI 89.35%-99.95%)
Positive predictive value	92.31% (95% CI 79.13%-98.38%)	92.73% (95% CI 82.41%-97.88%)	98.11% (95% CI 89.93%-99.95%)
Negative predictive value	65.28% (95% CI 53.14%-76.12%)	82.14% (95% CI 69.60%-91.09%)	84.48% (95% CI 72.58%-92.65%)
Likelihood ratio	9.83	10.45	42.62

WVA: whole viral antigen; rNP: recombinant nucleocapsid protein.

**Table 2:** Summarized ELISA results for control, asymptomatic, and symptomatic groups. The values represent the number of samples obtained by comparing the WVA ELISA results and the rNP ELISA results.

Tests	Control	Asymptomatic	Mild symptoms	Moderate symptoms
WVA+/rNP +	01	03	25	07
WVA-/rNP -	44	05	04	0
WVA+/rNP -	02	0	01	0
WVA-/rNP +	03	0	13	03
Total	50	08	43	10
